# Malignant Syphilis Mimicking Lymphoma in HIV: A Challenging Case and a Review of Literature Focusing on the Role of HIV and Syphilis Coinfection †

**DOI:** 10.3390/microorganisms13050968

**Published:** 2025-04-24

**Authors:** Chiara Maci, Diana Canetti, Chiara Tassan Din, Elena Bruzzesi, Maria Francesca Lucente, Flavia Badalucco Ciotta, Caterina Candela, Maurilio Ponzoni, Antonella Castagna, Silvia Nozza

**Affiliations:** 1 School of Medicine, Vita-Salute San Raffaele University, 20132 Milan, Italy; 2 Infectious Diseases Unit, IRCCS San Raffaele Scientific Institute, 20132 Milan, Italy; 3 Pathology Unit, IRCCS San Raffaele Scientific Institute, 20132 Milan, Italy

**Keywords:** syphilis, secondary syphilis, HIV pathogenesis, opportunistic infections, sexually transmitted infections

## Abstract

The rate of reported syphilis cases is increasing worldwide, particularly among men who have sex with men. In this scenario, malignant syphilis is a rare, severe form of secondary syphilis, typically observed in immunocompromised individuals and characterized by rupioid skin lesions, together with systemic symptoms that could lead to potentially life-threatening complications. We report the complex case of a 42-year-old man, previously diagnosed with HIV infection, presenting with a five-day history of fever and multiple lymphadenopathies. His immunovirological status was well controlled, and he was fully adherent to antiretroviral therapy. His clinical presentation was severe and ambiguous, with neurological involvement being progressively excluded. The diagnosis was confirmed by serological tests, while histopathological examination of an excised lymph node revealed disrupted architecture with multiple granulomas. Differential diagnosis, including lymphoma and other potential etiologies, was performed. After completion of antibiotic therapy, clinical symptoms completely resolved. No Jarisch–Herxheimer reaction occurred. We also provide an updated review of the current literature, with a focus on HIV coinfection, which is frequently associated with the development of malignant syphilis, and discuss the need for enhanced interventions to prevent sexually transmitted infections, as well as the importance of judicious use of doxycycline post-exposure prophylaxis.

## 1. Background

Sexually transmitted infections (STIs) are among of the most common infections and represent a global public health threat. Their incidence is rising steadily, particularly within members of key populations, such as men who have sex with men (MSM). Between 2021 and 2022, the trend of STIs diagnoses has notably grown among young people, and the number of reported cases of gonorrhea, syphilis, and chlamydia increased by 48%, 34%, and 16%, respectively [1]. In the United States, the Centers for Disease Control and Prevention (CDC) documented approximately 176,000 syphilis cases in 2021, contributing to an estimated 6 million new diagnoses worldwide.

In this scenario, malignant syphilis (MS) is a rare, severe form of secondary syphilis, which typically affects immunocompromised individuals. It is characterized by rupioid skin lesions, together with systemic symptoms that can lead to potentially life-threatening complications. We report a case of persistent fever and enlarged multiple lymph nodes caused by malignant syphilis mimicking lymphoma in a person with HIV infection, followed by a literature review focusing on severe variants of syphilis.

This paper was written according to the CARE guidelines: consensus-based clinical case report guideline development [2].

## 2. Case Presentation

A 42-year-old man was admitted to the Infectious Diseases Unit of the IRCCS San Raffaele Scientific Institute in Milan, Italy, in April 2023 with a three-month history of recurrent five-day fever, responsive to nonsteroidal anti-inflammatory drugs (NSAIDs), together with widespread, variably sized skin lesions, neither pruritic nor painful, and multiple, tender lymphadenopathies.

In October 2018 he was diagnosed with HIV (CDC stage A2), probably during an acute infection, with baseline undetectable viremia, a CD4+ count of 293 cell/µL, a CD8+ count of 739 cell/µL, and a CD4+/CD8+ ratio 0.4. He started antiretroviral therapy with a three-drug regimen based on dolutegravir/abacavir/lamivudine (DOL/ABC/3TC), rapidly achieving virological suppression and normal immunological status; the most recent blood test showed CD4+ 450 cell/µL, CD8+ 638 cell/µL, CD4+/CD8+ ratio 0.71, and undetectable HIV RNA.

His past medical history also included, since 2015, ulcerative colitis, treated with mesalamine 800 mg/day, and arterial hypertension, treated with bisoprolol. During the same year, he experienced hospitalization for myocarditis. In July 2022 he underwent multiple dental extractions.

Regarding STIs, he had a prior episode of *Chlamydia trachomatis* urethritis, appropriately treated with doxycycline, and underwent Mpox vaccination in 2023.

Finally, since March of the same year, he noted unintentional weight loss (6 lb/month) and profuse nocturnal sweating, even in the absence of fever. He denied recent travel to tropical regions and animal exposure.

### 2.1. General Physical Examination and Laboratory Test Results

At first medical evaluation, his vital parameters were normal, with blood pressure (BP) of 118/67 mmHg, heart rate (HR) of 97 beats per minute, and oxygen saturation level of 95%. He was febrile, confused, and not completely temporally and spatially oriented. Oral mucous membranes were normohydrated and intact, without lesions. His skin presented extensive multiple diffuse nodulopapular and pustular lesions on the upper limbs, trunk, and soles of the hands and feet, particularly concentrated in the genital region at the scrotum and penile shaft, with ulceration, that were neither itchy nor painful. The right inguinal lymph node, with a diameter of 2.5 cm, was mobile, tense, and painful, with additional ipsilateral supraclavicular, left cervical, and left occipital lymphadenopathies. Cardiac exam revealed a rhythmic heart rate and a systolic murmur 3/6 (already known) in tricuspid focus radiating to pulmonary focus. Thoracic conformation was normal, and vesicular murmur was perceived on lung fields, without added pathological noises. Peristalsis was present, valid, and not painful, including on palpation, Murphy’s sign was positive, and Blumberg’s sign was negative. Anogenital secretions were absent. In the lower limbs there was no edema or signs of deep vein thrombosis.

Upon admission, blood tests showed increasing inflammation markers with white blood cell (WBC) 10 × 10^9^ cells/L (75% neutrophils), mild anemia with hemoglobin (Hb) 12.5 g/dL, elevated C-reactive protein (CRP) 66 mg/L, normal liver function, and a slight increase in alkaline phosphatase (ALP) 194 U/L.

### 2.2. Diagnostic Methods

#### Microbiological Tests and Other Findings

A complete screening for STIs was performed. Serological tests for syphilis showed a positive rapid plasma regain (RPR) at a titer of 1:128, as well as *T. pallidum* particle agglutination assay (TPHA) at a titer of >1:5120. Oral and anal swabs for *Neisseria gonorrhoeae* and *Chlamydia trachomatis*, as well as serology for hepatitis, were negative. Blood and urine cultures were negative. Screening for anal cancer with cytology and HPV test were negative too.

### 2.3. Imaging Studies

**Figure 1:**
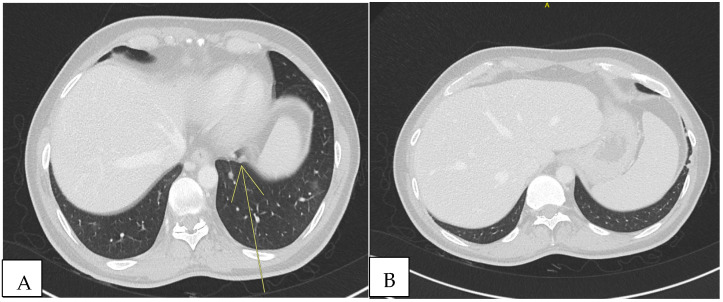
Total-body CT scan revealed non-specific pulmonary consolidations. Other diseases could explain these observations, such as sarcoidosis, neoplasm, or other infectious diseases (TB, NTM, herpesviruses, cryptococcosis). (**A**) Nodule in retrocardiac area (yellow arrow). (**B**) Two nodules in subpleural area.

Because of the positive Murphy’s sign, he underwent abdominal ultrasound, which detected hepatomegaly without focal lesions. A total-body computed tomography (CT) scan revealed multiple supra- and infra-diaphragmatic lymphadenopathies, some with colliquative features, with the largest left cervical measuring 31 mm × 16 mm. In the lung lobes, there were few bilateral nodular formations with prevalent subpleural distribution, measuring 6 to 8 mm. In the left lower lobe, located paracardially, parenchymal thickening with elongated morphology and pseudo-nodular components were detected. In the same areas, shaded areas of increased parenchymal density, predominantly parabronchial, suspicious for small airway inflammation, were also described (Figure 1).

### 2.4. Neurological Tests and Imaging Studies

Since at admission the patient was confused and agitated, brain CT and magnetic resonance imaging (MRI) scan were performed to rule out any abnormalities. The persistence of symptoms led to investigating the potential occurrence of neurosyphilis, but the cerebrospinal fluid (CSF) analysis showed four cells, with negative cultural exams and PCR, while TPHA came back negative about 15 days later.

### 2.5. Otorhinolaryngological Consultation

He also underwent otolaryngologist evaluation and was diagnosed with acute pharyngotonsillitis, without additional pathological findings.

### 2.6. Differential Diagnosis and Histopathological Results

**Figure 2:**
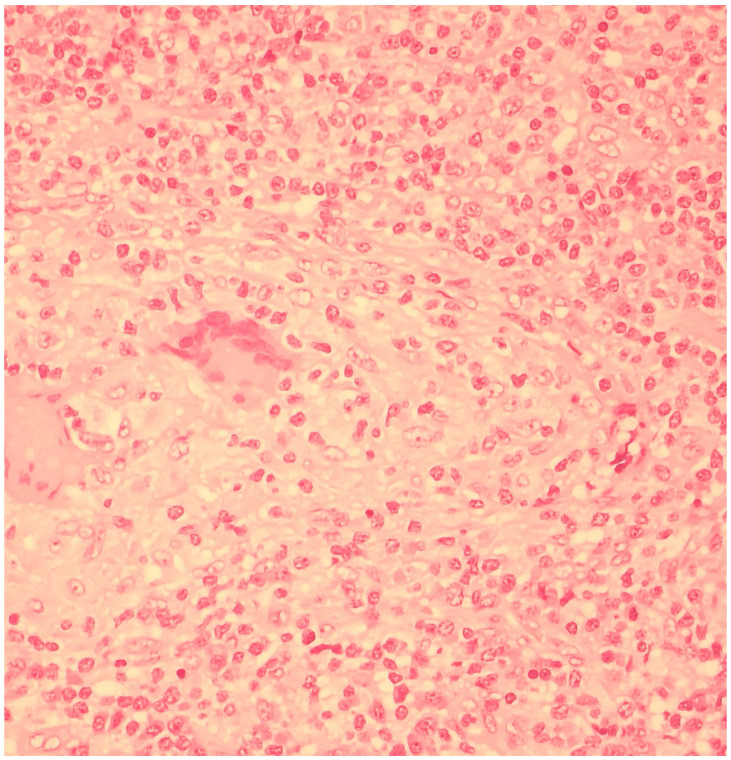
Histopathological examination of the left cervical lymph node showed partial effacement of the lymph node architecture due to the presence of non-necrotizing granulomas containing multinucleated giant cells. The anti-*Treponema* immunoreaction was negative.

The severe clinical presentation prompted the exclusion of other infectious and non-infectious diseases. The QuantiFERON-TB Gold test was negative, as well as CMV-DNA and EBV-DNA quantitative assessment of plasma and serology for *Bartonella*. Histopathological investigations were performed to further exclude oncological and hematological etiologies. For this reason, diagnostic surgical excision of the left cervical lymph node was carried out, and histopathological examination showed partial effacement of the lymph node architecture due to the presence of non-necrotizing granulomas containing multinucleated giant cells. In the interfollicular area, a moderate number of mature-looking plasma cells and collections of histiocytes with abundant and clear cytoplasm were found (Figure 2). Direct immunofluorescence test for *Mycobacteria* was negative, as well as the anti-*Treponema* immunoreaction. The additional presence of opportunistic microorganisms or neoplastic cells was not demonstrated.

### 2.7. Treatment

The patient was treated with intravenous penicillin-G therapy for 14 days, rather than with one intramuscular administration of benzathine penicillin-G in a single dose. Since we could not rule out neurosyphilis based on the blood analysis results, and given the neurological manifestation upon hospital admission, the patient was treated more extensively.

### 2.8. Jarisch–Herxheimer Reaction

Before administration of intravenous penicillin-G, the patient started treatment with amoxicillin/clavulanate, awaiting diagnostic confirmation. After starting penicillin-G, he did not experience worsening of symptoms or the Jarisch–Herxheimer reaction.

Therapy was temporarily discontinued for 24 h on day 7, due to intolerance of the antibiotic treatment, with subsequent onset of fever, that was well managed with paracetamol, and the antibiotic was resumed.

### 2.9. Outcome and Follow-Up

By day 14, he experienced progressive clinical improvement, the fever and confusion resolved, the lymphadenopathies regressed, and inflammatory markers normalized. Serological follow-up was performed, until the RPR titer was reduced to 1:2. Afterwards, the patient underwent screening at our center every 6 months. At the end of treatment, neck ultrasound showed a bilateral reduction in the size of the cervical lymph nodes, and the skin lesions had disappeared.

After 10 months, fludeoxyglucose F18 (18F-FDG) positron emission tomography (PET)/CT scan was performed, showing regression of the lung consolidation.

## 3. Definition

Also known as lues maligna, MS is a severe and widespread form of secondary syphilis that primarily occurs in immunocompromised individuals, such as those coinfected with human immunodeficiency virus (HIV). This form of syphilis is a systemic manifestation of disease and represents the stage at which the bacteria have spread into the bloodstream and reached their maximal amount. This stage of syphilis affects about 25% of people with untreated infection. It occurs within a few weeks to a few months after development of the primary infection. However, patients with secondary syphilis may not have a history of previous syphilis, because the primary infection may have been asymptomatic or undetected [3].

Similar to the primary disease, acute manifestations of secondary syphilis generally resolve spontaneously, even without therapy, except in cases of severe skin ulcerations. Occasionally, untreated individuals experience relapsing episodes of secondary disease, which may occur up to five years later. Particularly, systemic clinical manifestations of MS can be several, with fever, headache, fatigue, and myalgia, typically associated with papular-pustular skin lesions, characterized by necrotic evolution. They evolve in well-demarcated ulcers with brown hemorrhagic crusts arranged in concentric, rupioid layers, often sparing the palms and soles, as documented in our case.

## 4. Epidemiology

Between 2020 and 2021, syphilis cases increased globally, particularly by 4% among MSM [4]. A substantial increase has been also observed in high income countries. In 24% of cases of untreated syphilis, the disease may manifest as asymptomatic early or late latent syphilis or present with one or more manifestations as secondary syphilis [5,6].

In the ECDC Report 2023, 29 EU/EEA member states reported 41,051 confirmed cases of syphilis. The incidence is seven-fold higher in men than in women, with 77% reported cases with transmission tracking information comprising MSM.

Also, in 2023, 52% cases of syphilis among people with HIV (PWH) were described by 16 countries [7].

In HIV coinfected individuals, the incidence of malignant syphilis is increasing, initially standing at 0.36%, now assessed between 1.2 and 3.8% by more recent studies [8,9,10].

Estimates of trends in incidence and overall impact on the health system are essential for syphilis prevention strategies. Over the last decade, a substantial increase in syphilis has been observed among people in higher income countries. These data highlight the need for further public health interventions and strategies, where the groups at greatest risk of infection are MSM. Furthermore, increased incidence was documented among women of reproductive age, especially during pregnancy. Additionally, early diagnosis and prompt treatment of syphilis are crucial in pregnant women, where consequences can be catastrophic [11].

For MSM and PWH, screening should be—at least—offered annually or more frequently if the risk is particularly high [12,13]. Although the optimal interval has not been established, screening should be considered in all asymptomatic, non-pregnant adolescents and adults who are sexually active with consequent high risk for syphilis infection.

## 5. Demographic Features

Most patients are male, with a median age of 41 years [14].

Specifically, reported syphilis rates are eight times higher in men than in women, with the highest rate of diagnosis among 25- to 34-year-old men. In Europe, the incidence in women is 1.9 cases per 100,000 population [1]. In cases where maternal infection is not detected and treated early enough in pregnancy, maternal-fetal transmission has several important consequences. The prevalence ranges between 1.4% and 2.9% [15,16].

Additionally, diagnosis is more frequent among MSM, with an overall incidence of 76.4 per 1000 person-years globally, with notable variations by region and country [17]. Finally, the prevalence of syphilis in transgender women (TGW) tested in several studies ranged between 17% and 38% [18,19,20,21].

## 6. Clinical Characteristics

Generally, syphilis is characterized by wide-ranging clinical features, with three main clinical patterns—primary, secondary, and tertiary—often based on individuals’ immune status.

Infection becomes detectable about 21 days after exposure. Primary syphilis is represented by localized skin lesion called “chancres”, potentially associated with regional lymph node involvement. About 25% of people with untreated infection, regardless of the presence of a primary lesion, develop a systemic illness, secondary syphilis, within weeks to a few months after chancre develops. At this stage, clinical manifestations are various, ranging from constitutional symptoms such as fever, erythematous rash, headache, fatigue, myalgia, and lymphadenopathy to more specific and severe manifestations with abdominal, neurological, ocular, and otovestibular involvement. Among immunocompetent individuals, the acute manifestations of secondary syphilis typically resolve spontaneously, even without treatment, except in severe forms of MS. Occasionally, untreated individuals experience relapsing episodes of secondary disease, which may occur up to five years later.

Late syphilis occurs in about 25% to 40% of untreated patients 1 to 30 years after primary infection, regardless of past clinical manifestation [22]. It can be characterized by cardiovascular involvement, especially with aortitis, rare gummatous form with granulomatous, nodular lesions, potentially on several organs, but commonly on skin and bones, and central nervous system (CNS) symptoms, especially with generalized paresis and tabes dorsalis.

In the 15th century, syphilis was already described, and several cases were later reported over the centuries. Precisely, MS was firstly described in 1897 by the German physician Albert Ludwig Sigesmund Neisser, who defined the main typical features of this severe form of secondary syphilis and its evolution: short incubation period, prodromal symptoms, pleomorphic skin lesions, nodular lesions on mucous membranes, and mild mucous patches [23,24].

The incubation period can vary from 6 weeks to 1 year, although it is usually shorter. The prodrome symptoms are characterized by fever, myalgia, significant asthenia, headache, and photophobia, anticipating the eruption of lesions.

Then, skin lesions appear with well-defined, raised borders and central necrosis and may develop crusting consisting of brown or black lamellar plaques, which often require appropriate treatment for removal. In addition, skin nodules can be associated with evolution of the ulcerative appearance. Based on these characteristics, the so-called “rupioid lesions” derive their name from the Ancient Greek word “rhupo” (ῥύπος), meaning “dirt”. They are most frequently seen on the scalp and head, but they can also affect the trunk and extremities, with palms and soles often spared [25]. The plasma cell–rich mononuclear infiltrate reflects an immunological response; however, the pathogenesis of the skin lesions of secondary syphilis is still unclear. In our case, *T. pallidum* was not found in the lesions, but the histological infiltrate was typical, and the characteristics of the histopathological samples were consistent with the diagnosis. Other recent studies showed the presence of *T. pallidum* at the dermo-epidermal junction, suggesting that skin lesions of malignant syphilis may be caused by spirochete direct invasion [26]. Involvement of the mucous membranes, including mouth and genitals, is usually less common.

Concurrently, as in secondary syphilis, in MS other several organs can be involved, such as with generalized lymphadenopathy, ocular scleral nodules or keratitis, and leonine facies. The most frequent involvement results in bone and joint localization, with pain typically reported. Also, approximately 50% of cases present CSF alterations, even without neurological symptoms. Criteria for diagnosis are currently based on laboratory findings and/or combined with clinical manifestations. However, it is preferable to treat patients with neurosyphilis in the asymptomatic phase to avoid serious and irreversible sequelae [27].

The ears can also be involved, commonly with vestibular disorders, sometimes leading to sensory-neural hearing loss in both ears simultaneously or sequentially [28,29]. The spirochetes can involve the vascular system with several manifestations, for instance syphilitic aortitis, which may lead to subsequent aortic regurgitation and heart failure. However, osteitis and arthritis are rarely described [30]. Additionally, pulmonary involvement is extremely rare in patients with secondary syphilis. However, as our clinical case showed, lung involvement may manifest as infiltration, consolidation with pleural effusion, or solitary or multiple pulmonary nodules [31]. Even though the prevalence of kidney involvement varies between 0.3% and 0.8%, its main clinical manifestation is nephrotic syndrome, in particular membranous nephropathy, acute kidney injury, membranoproliferative glomerulonephritis, and interstitial nephritis [32].

## 7. Diagnosis

Criteria for diagnosis are based on laboratory findings and/or combined with clinical manifestations. Syphilis is diagnosed by the determination of non-treponemal antibodies (i.e., rapid plasma regain/RPR, VRDL), which also allow a quantitative value to be defined, and confirmed by specific treponemal antibodies (TPHA, FTA-Abs, ELISA). Non-treponemal tests are based on cardiolipin and are also known as RPR or VDRL. These tests are still commonly used as screening tests; they are cheap and easy to perform. The advantages of treponemal tests are their high sensitivity and specificity. Indeed, TPHA is generally accepted as the most sensitive and is considered the gold standard diagnostic method. After treatment, the titer of nontreponemal tests decreases and becomes negative, whereas treponemal tests remain positive throughout the lifespan [33].

The criteria used to define malignant syphilis are the presence of rupioid lesions, the involvement of visceral organs, the Jarisch–Herxheimer reaction, and the histological pattern [34,35]. Few or no spirochetes can be detected by Steiner or Warthin–Starry cytochemical stains, or by immunohistochemistry, and the most common lesion type relies on plasma cell infiltrate, occasionally accomplished by granulomas. In addition, other organ involvement must be investigated, and CNS, ocular, or hearing involvement must be promptly excluded to determine the correct treatment. Neurosyphilis in MS occurs more frequently than in cases of secondary syphilis. This unusual combination cannot be considered a simple coincidence, but a new phenomenon that is emerging and needs to be further investigated with more substantial clinical data in the context of the current syphilis epidemic. Also, HIV coinfection could alter the natural history of syphilis and increase the risk of developing neurosyphilis, which is why it is recommended to perform lumbar puncture (LP) in PWH [8,27,36].

## 8. Differential Diagnosis

Syphilis has been called the “great imitator” because of its heterogeneity and ability to mimic malignant diseases and other conditions presenting with similar cutaneous manifestations, including ulcerative pyoderma, chronic pityriasis lichenoid, and acute varicelliform versicolor [37]. In particular, the frequent and significant lymph node involvement may raise suspicion of lymphoma, such as T-cell lymphoma, especially since PWH are more susceptible to malignancy, probably due to immune dysfunction [38,39,40]. When pulmonary nodules of undetermined significance are associated with generalized lymphadenopathies, disorders like sarcoidosis, or other infectious diseases (NTM, herpesviruses, cryptococcus) should be excluded. Regarding rheumatological diseases, the several clinical presentations of MS may overlap with Behçet’s disease, a systemic inflammatory disease affecting multiple systems, including ocular inflammation, arthritis, oral and genital ulcers, skin lesions, and visceral organ involvement [41].

Histopathological differential diagnosis encompasses granulomatous disorders (including mycobacteriosis and leprosy), vascular infiltrates with leukocytoclastic vasculitis, and plasma cell–rich disorders, including HHV8 infection or IgG4-related disease. This does not exclude other diseases such as necrotizing generalized herpes zoster or Mpox [42,43].

## 9. Treatment

Following CDC guidelines, the therapy for malignant syphilis is the same as that for secondary syphilis, which is benzathine penicillin G 2.4 million units IM in a single dose. However, in patients allergic to beta-lactams, the therapy is doxycycline 100 mg every 12 h for 14 days. In forms of severe clinical involvement of secondary syphilis, it is important to exclude neurological, ocular, or auricular involvement, as then it is considered tertiary syphilis, and therefore treatment includes aqueous crystalline penicillin G 18–24 million per day as a continuous infusion or, in refractory cases, extended infusion for 4 h a day for 10–14 days [44]. In cases of doubtful neurological involvement or delayed CNF analysis results, it is preferable to treat patients with suspected neurosyphilis promptly to avoid serious and irreversible sequelae [27].

## 10. Malignant Syphilis in Immunocompetent Hosts

MS more frequently occurs in conditions of cell-mediated immunity dysfunction, which is rare among immunocompetent individuals. The pathogenesis of MS is unknown, but it can be the result of immunosuppression, inappropriate immune response, and a virulent strain of *Treponema pallidum*. It is generally believed that HIV coinfection makes *T. pallidum* more virulent. Indeed, the incidence of MS in PWH appears to be 60 times higher [26]. However, since the incidence of syphilis is increasing, cases of malignant syphilis are also on the rise in immunocompetent individuals. A systematic review of MS revealed that 6 of 12 HIV-negative patients (50%) had comorbidities such as diabetes mellitus, alcoholism, drug abuse, psoriasis, and hepatitis, which could affect their immune function. These findings highlight the correlation between MS and other co-morbidities potentially related to an aberrant immune response, leading to severe skin manifestations, together with virulent strains of *T. pallidum* [14].

Cases of MS in individuals who are receiving immunosuppressive/immunomodulatory treatment also deserve attention, with a few cases of MS associated with anti-tumor necrosis factor (TNF), adalimumab, or infliximab reported in the literature. A case of MS in a patient with Crohn’s disease treated with the anti-interleukin-6 tocilizumab and another case in a patient with Waldenström’s macroglobulinemia treated with ibrutinib were also reported in the current literature [45,46]. As previously mentioned, a stronger association between MS and neurosyphilis is emphasized, regardless of immunological status and even in the absence of HIV infection [26].

## 11. Malignant Syphilis in People with HIV

Syphilis is more common among PWH, with new cases increasing over the time. The incidence rate of syphilis is 4.7 cases per 100 person-years, and, among MSM, the rate of coinfection with HIV and syphilis ranges from 30% to 60%, depending on geographical location [47].

PWH are approximately 60 times more likely to develop MS after the appearance of asymptomatic chancres or secondary or latent infection. In individuals where viral suppression is not achieved, a consequent higher incidence of new and recurrent syphilis is recorded compared with those who are virologically suppressed [48,49]. As well as other coinfections, such as tuberculosis or herpes simplex, syphilis can have a negative impact on the immunological and virological status of PWH. HIV RNA levels increase during primary and secondary syphilis, potentially leading to a greater risk of HIV transmission. In terms of immunological status, the levels of total lymphocytes, CD4+ T cells, and CD8+ T cells decrease during syphilis infection and recover after treatment [36,37,38,39,40,41,42,43,44,45,46,47,48,49,50,51].

The onset of symptoms of secondary syphilis does not differ between people with or without HIV, usually occurring 3 to 6 weeks after resolution of the primary stage. The hematogenous dissemination of treponemes in the early stages of infection can lead to the widespread and severe consequences of secondary syphilis, including dermatological, neurological, and ocular manifestations. There are several reported cases of PWH with neurosyphilis at clinical presentation, showing that early neurological involvement may be more common in PWH. Possible risk factors for the development of neurosyphilis include CD4+ count below 350 cells/μL, rapid plasma reagin titer >1:128, and male sex. Although ocular syphilis occurs in both people with and without HIV, it has been reported more frequently in PWH [52,53]. Considering this evidence, it is recommended to regularly test PWH with risk factors for STIs for syphilis, so that it can be promptly treated, avoiding more serious clinical conditions.

## 12. DoxyPEP and Syphilis

With the increase in syphilis cases and the consequent rise in MS, it is important to understand the crucial role of post-exposure prophylaxis for STIs. According to a recent International Union Against Sexually Transmitted Infection (IUSTI) position paper, the use of doxycycline as post-exposure prophylaxis (doxyPEP) is recommended for people at high risk of STIs. The decision to offer doxyPEP must be guided by the patient’s risk factors, the local epidemiology of STIs, and the potential impact on local public health. The impact of doxyPEP on local and regional antimicrobial resistance patterns, both for STIs pathogens and for other bacterial infections, could also be assessed. Furthermore, periodic screenings for STIs and vaccine prophylaxis should be offered despite the use of doxyPEP [54,55].

According to recently published studies, a reduction in the incidence of STIs has been demonstrated, with less impact on gonorrhea due to the increasing rate of tetracycline resistance [56,57]. Regarding syphilis, although the incidence is lower than other STIs, a reduction in syphilis cases in those who regularly take doxyPEP compared to those who do not has been demonstrated [58,59].

## 13. Conclusions

Syphilis, known as “the great imitator”, presents with several, diverse, and often misleading clinical manifestations, and MS is an even trickier diagnostic challenge due to its variability.

The rise in syphilis cases at all stages over the past 5 years has brought attention to a disease that appeared to be well-known and under control.

This article presents a case of MS that clinically imitated a lymphoma, suggesting how crucial is to consider differential diagnosis with autoimmune, hematological, or other infectious diseases. When interpreting clinical presentations, this approach is essential to avoid missed diagnoses and delayed treatment. Furthermore, the rational use of doxyPEP could reduce the risk of STIs and is also well accepted among MSM and TGW [60].

## Data Availability

Data are not publicly available, but are available from the corresponding authors on reasonable request (maci.chiara@hsr.it; nozza.silvia@hsr.it).
